# Piloting a new method to estimate action thresholds in medicine through intuitive weighing

**DOI:** 10.1136/bmjebm-2023-112350

**Published:** 2023-08-30

**Authors:** Bart K M Jacobs, Alfred Kipyegon Keter, Aquiles Rodrigo Henriquez-Trujillo, Paco Trinchan, Madeleine L de Rooij, Tom Decroo, Lutgarde Lynen

**Affiliations:** 1 Department of Clinical Sciences, Institute of Tropical Medicine, Antwerpen, Belgium; 2 Department of Applied Mathematics, Computer Science and Statistics, Ghent University, Ghent, Belgium; 3 Human Sciences Research Council, Sweetwaters, Pietermaritzburg, South Africa; 4 Facultad de Medicina, Universidad de Las Américas, Quito, Ecuador; 5 Health Services Department, Bulawayo City Council, Bulawayo, Zimbabwe

**Keywords:** Clinical Decision-Making, Evidence-Based Practice, Methods, Tropical medicine, Policy

## Abstract

**Objectives:**

In clinical decision-making, physicians take actions such as prescribing treatment only when the probability of disease is sufficiently high. The lowest probability at which the action will be considered, is the action threshold. Such thresholds play an important role whenever decisions have to be taken under uncertainty. However, while several methods to estimate action thresholds exist, few methods give satisfactory results or have been adopted in clinical practice. We piloted the adapted nominal group technique (aNGT), a new prescriptive method based on a formal consensus technique adapted for use in clinical decision-making.

**Design, setting and participants:**

We applied this method in groups of postgraduate students using three scenarios: treat for rifampicin-resistant tuberculosis (RR-TB), switch to second-line HIV treatment and isolate for SARS-CoV-2 infection.

**Interventions:**

The participants first summarise all harms of wrongly taking action when none is required and wrongly not taking action when it would have been useful. Then they rate the statements on these harms, discuss their importance in the decision-making process, and finally weigh the statements against each other.

**Main outcome measures:**

The resulting consensus threshold is estimated as the relative weights of the harms of the false positives divided by the total harm, and averaged out over participants. In some applications, the thresholds are compared with an existing method based on clinical vignettes.

**Results:**

The resulting action thresholds were just over 50% for RR-TB treatment, between 20% and 50% for switching HIV treatment and 43% for COVID-19 isolation. These results were considered acceptable to all participants. Between sessions variation was low for RR-TB and moderate for HIV. Threshold estimates were moderately lower with the method based on clinical vignettes.

**Conclusions:**

The aNGT gives sensible results in our pilot and has the potential to estimate action thresholds, in an efficient manner, while involving all relevant stakeholders. Further research is needed to study the value of the method in clinical decision-making and its ability to generate acceptable thresholds that stakeholders can agree on.

**WHAT IS ALREADY KNOWN ON THIS TOPIC**

- Many methods for estimating action thresholds exist but few are adopted in clinical practice and no gold standard exists.

**WHAT THIS STUDY ADDS**

- We piloted a method that provides sensible estimates of action thresholds which, through its form, has the potential to be more easily adopted in clinical decision-making.

**HOW THIS STUDY MIGHT AFFECT RESEARCH, PRACTICE OR POLICY**

- Better evidence-based action thresholds that are adopted in clinical practice and guideline development will lead to better practices and can save valuable resources.

## Introduction

### The threshold approach in clinical decision-making

When confronted with diagnostic uncertainty clinicians will usually intuitively weigh the harm and the benefit of the possible actions they can take. For example, when seeing a patient who may have a curable disease, treatment will only be offered when one is sufficiently certain that the benefit of treatment outweighs the harm and consequences of leaving the disease untreated. This reflects how confident clinicians want to be about the patient having the disease before taking action. For a lethal disease where quick action is necessary, such as malaria, clinicians may choose to treat it even when malaria is not very probable and alternative diagnoses require less urgency. Conversely, for ailments where the treatment is very toxic or otherwise imposes a serious burden on the patient, they want near 100% certainty to start treatment, for example, when chemotherapy in a patient with a suspicious lesion in the lung is being considered. In both cases, physicians will prescribe the treatment only when the probability of the disease is above a certain intuitive threshold.^1^

Such a threshold approach is not unique to medicine. It was first introduced in the medical field through the concept of the therapeutic threshold: the probability of disease at which the decision on whether or not to start treatment is at equipoise.^2^ It takes into account the net harm and benefit of false positives, false negatives, true positives and true negatives.

However, action thresholds in medicine are not always therapeutic thresholds. In the suspicious lung lesion example, no clinician would immediately prescribe chemotherapy but instead consider further testing. Each possible test, such as a scan or a biopsy, has its own action threshold, called the test threshold. The test thresholds can either be derived directly from the therapeutic threshold based on the diagnostic accuracy of the test or estimated separately, especially when the test itself imposes possible harm or cost, for example, in the case of invasive tests.

Examples of other action thresholds in medicine can relate to changing medication or other types of treatment, treatment interruption (due to toxicity or futility, or getting into palliative care), isolation or quarantining of a patient, or the decision to hospitalise or transfer a patient to a different ward (eg, transfer from/to the intensive care unit).

### Knowledge gap in threshold models

To make clinical decisions in a sensible way, it is key to have precise estimates of relevant action thresholds. However, in practice, most decisions do not explicitly include this thought process. While several methods to estimate action thresholds exist, few give satisfactory results or have been adopted in clinical practice. To the best of our knowledge, no consensus exists on the best approach to estimate the thresholds.^3 4^

Prescriptive methods aim to quantify the harms and benefits based on theoretical models. These are in essence all based on von Neumann-Morgenstern utility theorem, and therefore, consider an idealised world.^5^ However, expected utility theory can only include objective quantifiable harms (mortality, cost, morbidity, …). Unsurprisingly, this approach has been shown to produce results that do not match with physicians’ actual choices in practice. Some other prescriptive methods also try to include subjective quantifiable sources of harm (regret, fear of retaliation, etc) to offset this difference.^3 6–9^

Descriptive methods conversely seek to understand and predict how actual decision-makers behave.^10^ They, therefore, do not try to estimate harms directly but instead derive the threshold from observing clinical practice or a surrogate thereof.^11–15^

Next to resulting in very different thresholds,^4 16^ one could also wonder whether these methods estimate the same threshold since prescriptive methods focus on a theoretical model where the known and quantifiable harms or utilities are weighed, while descriptive methods instead observe the actual, potentially guidelines-driven threshold that does not need to coincide with the theoretically optimal threshold.^4 10^

Additionally, local factors such as the availability of and patient access to equipment or approved medication are expected to influence action thresholds, meaning the threshold has to be estimated separately for each local setting. This process has to be repeated when underlying conditions change over time.

Therefore, we need a method that is easy and rapid to perform, gives reliable and accurate estimates of the different action thresholds and incorporates reflection and feedback from a representative group of local physicians and healthcare workers to enhance the acceptability of and compliance with the guideline during implementation.

## Methods

### Introducing adapted nominal group technique

During classes with postgraduate students, we piloted a new method which we called the adapted nominal group technique (aNGT). We hypothesised that a formal consensus method with its roots in social sciences would allow participants to formulate all the different harms they perceived to be associated with an action threshold and discuss the relative weights of all components.^17 18^ Such methodology was used previously to decide on an algorithm to develop targeted viral load testing.^19^

**Figure 1:**
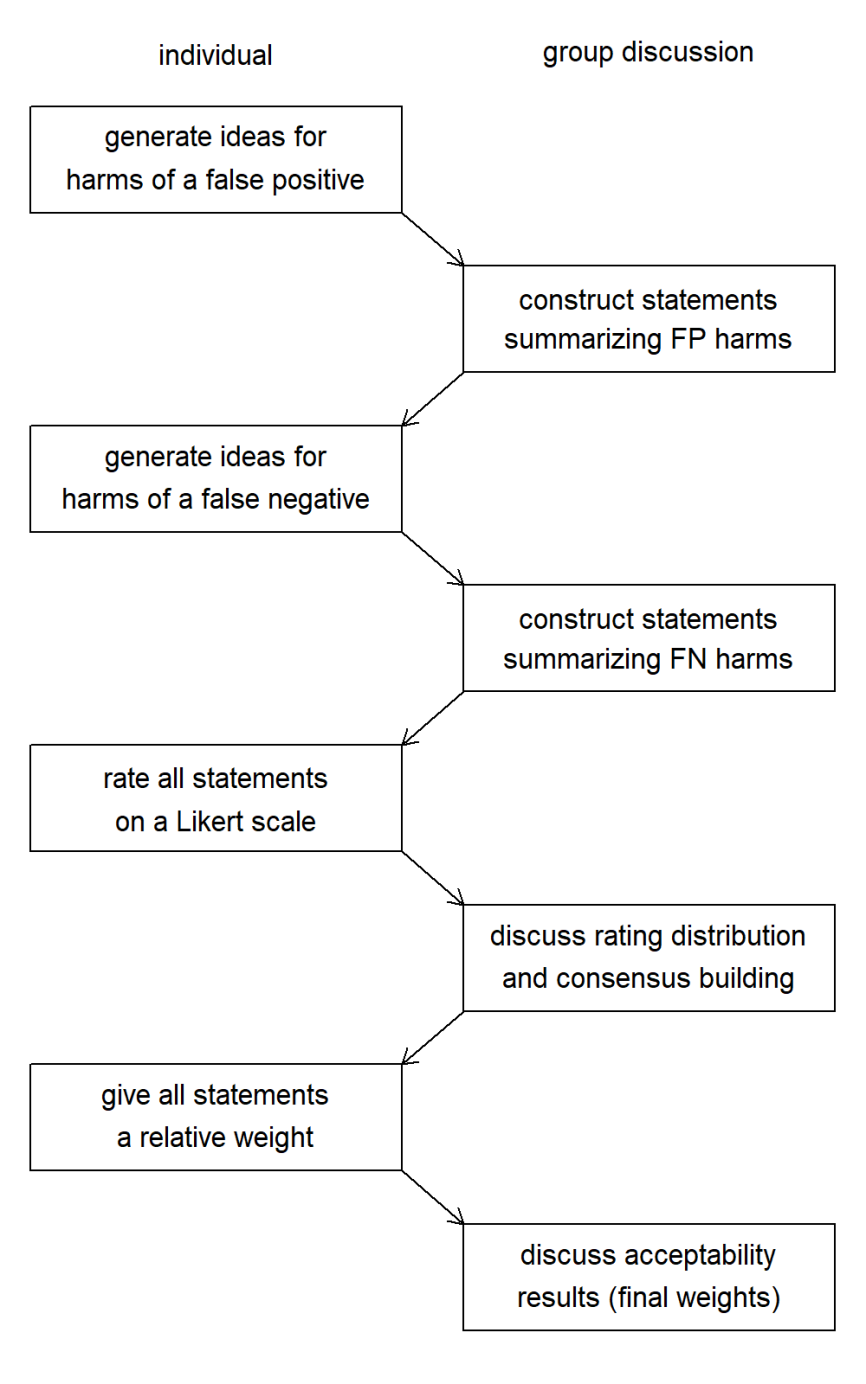
The different steps in adapted nominal group technique. The first steps can also be reversed in order, with harms of false negatives (FN) collected before the harms of the false positives (FP). The rating and weighing step could be repeated to check for consensus.

The steps are shown in figure 1. An example with all steps included can be found in online supplemental information. Our method follows the first steps of NGT, letting the participants to formulate ideas, in this case regarding the harms associated with making the wrong decision. These consist of two distinct sets, the so-called false positives: meaning unnecessary action, such as prescribing treatment or a test, was taken; and false negatives, meaning no required or beneficial action was taken. In practice, most decisions will result in so-called true positives and true negatives. However, the potential benefits of a true positive are already considered when the clinicians think on the potential harms of a false negative (the forgone benefits of a true positive). Similarly, the potential benefits of a true negative, if any, are considered when thinking about the harms of a false positive (eg, missing out on a correct differential diagnosis). Thus, all benefits from correctly taking or withholding action are implicitly included as harms, as every harm is a foregone benefit and any benefit is a foregone harm. Therefore, we only need to include the harms.

10.1136/bmjebm-2023-112350.supp1 Supplementary data

All harms are summarised and reformulated into clear statements in such a manner that (1) they are clearly understood by all participants and (2) they form a partition. The latter means that they are (2a) mutually exclusive, that is, there is no overlap between the harms and (2b) that to the extent possible, they jointly consider the total body of potential harm, that is, any harm reasonably associated with the action is included.

Next, the participants assess the importance of avoiding each harm in clinical practice when considering taking action by rating them on a Likert scale. Like NGT, we allow for discussion at this point and work towards consensus. Rerating of statements after the discussion can be considered to evaluate if a consensus was reached.

**Figure 2:**
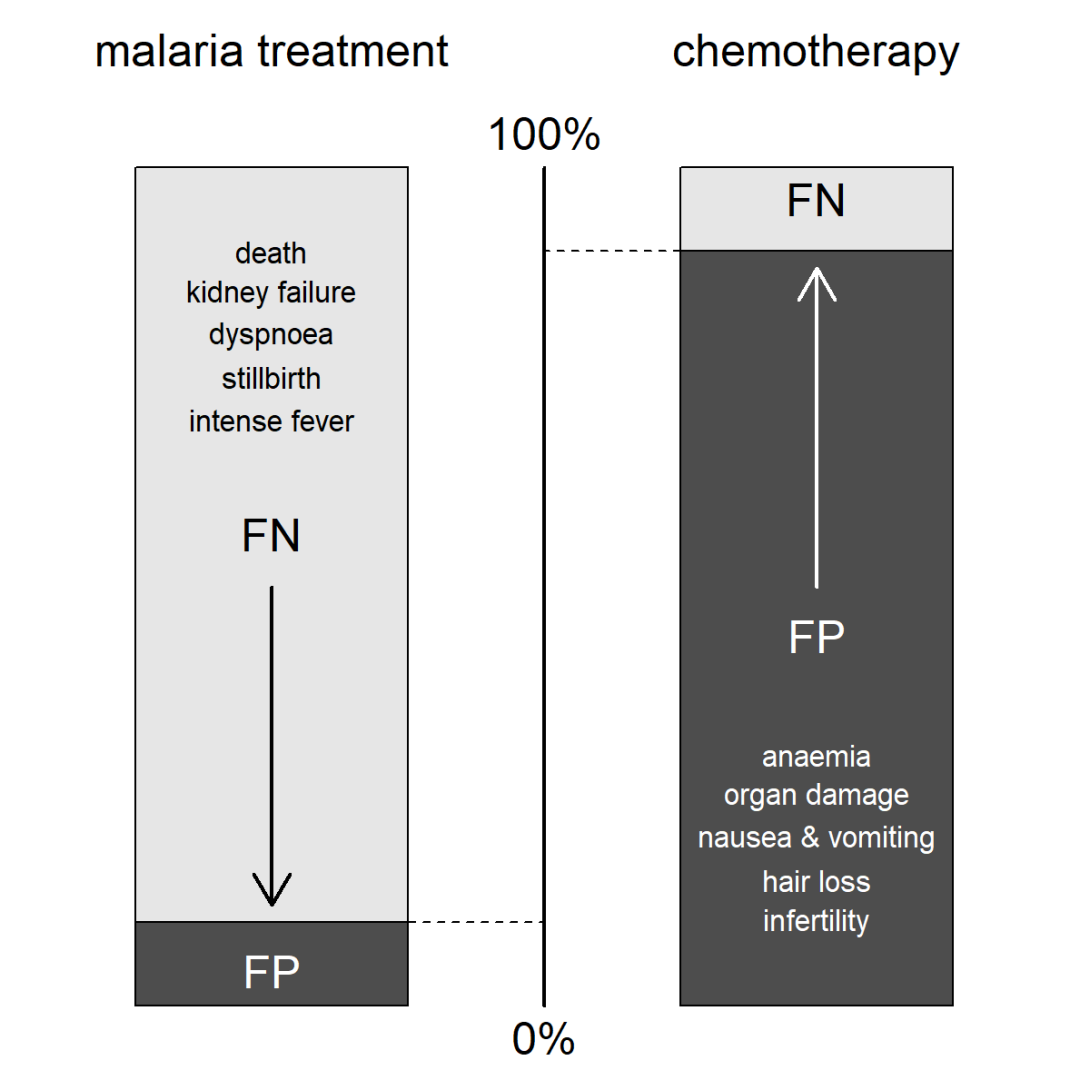
Examples of action thresholds. The action threshold is calculated as the relative percentage of harm caused by false positive diagnoses. If a false positive diagnosis leads to severe harm, this will push the threshold up as illustrated by the toy example on the right. If a false negative diagnosis induces major harm conversely, then the threshold will be pushed down as the relative percentage of harm of a false positive diagnosis is low as illustrated by the example on the left.

The key difference with classic NGT is that no statements are dropped, nor is it about ranking them. Instead, in the last step, participants weigh all harms against each other by distributing 100 (percentage) points over the different statements. The action threshold can then be estimated at the participant and panel levels by taking the (relative) percentage points assigned to the harms associated with false positives as illustrated in figure 2. Equivalently, the action threshold equals 100% minus the percentage points assigned to false negatives, as the total harm was fixed at 100%. This matches intuition since more harm caused by false positives will lead to a higher action threshold (the clinician must be more certain to take action), as illustrated by the example of considering chemotherapy, whereas higher harm from false negatives will lead to a lower action threshold (the clinician will take action even when not quite certain), as illustrated by the example of a potential malaria infection. Repeating the weighing of the statements after showing the results can be considered.

### Data collection

We piloted this method in seven groups of postgraduate students with experience in the healthcare sector on six different occasions. The main goal was educational; to introduce the concepts of weighing harms and action thresholds to students. The analysis presented here is a secondary use of fully anonymous data collected during these classes as part of the teaching activities. All responses were collected through the audience response platform Mentimeter.^20^ The following scenarios were used:

#### Rifampicin-resistant tuberculosis

We asked the students to consider, rate and weigh the harms associated with the decision to give a patient rifampicin-resistant tuberculosis (RR-TB) treatment versus treatment for rifampicin-susceptible TB (RS-TB). RR-TB treatment is known to have a longer duration and potentially be more toxic but is necessary in the case of RR-TB. However, drug susceptibility testing is imperfect and may not be available in some settings, meaning the treatment decision is often taken under uncertainty.^21^

This scenario was used twice in a face-to-face (F2F) setting, and once online, with students split into two smaller groups on the latter occasion for a total of four different aNGT sessions.

#### Treatment switching in HIV

Before viral load testing was readily available and affordable, the decision to start second-line treatment in people living with HIV in low- or middle-income Countries (LMIC) had to be taken under more uncertainty. We asked the students to consider, rate and weigh the harms associated with the decision to start second-line treatment versus continuing first-line treatment in a poor resource setting in the early 2000s. Second-line treatment was known to be more costly to both the patient and healthcare system and reduced future options if not justified, but was critical to avoid deterioration and opportunistic infections if resistance to first-line treatment had indeed occurred.^22^

This scenario was used once in an F2F setting and once online.

#### Isolation for COVID-19

Early in the COVID-19 outbreak, no reliable testing capacity was available. In this scenario, we asked the students to consider, rate and weigh the harms associated with isolating a person who is potentially infected with a newly discovered disease, now known to be SARS-CoV-2, the virus causing COVID-19. Isolation is known to severely impact people,^23^ while not isolating early patients could increase transmission and give rise to large numbers of COVID-19 patients and the associated pressure on the healthcare system.^24–26^

We used this scenario once, in an F2F setting.

#### Clinical vignettes

Because we wanted to demonstrate both prescriptive methods (such as aNGT) and descriptive methods to the students, we also developed clinical vignettes for RR-TB and COVID-19.^14 15^ These were filled in online by the students on two occasions linked to the RR-TB example, using an in-house platform, and in an F2F setting using Mentimeter for the COVID-19 example.

#### Facilitators and facilitator observations

All sessions were organised and attended by two or more facilitators: (1) at least one clinical scientist (MD with a specialised degree (master of public health or internal medicine)) and (2) at least one experienced biostatistician (Master and/or PhD). All facilitators are authors of this manuscript and all authors of the manuscript facilitated at least one aNGT.

The clinical scientist usually introduced the session, concepts and setting, moderated the discussion and responded to any relevant medical questions. The statistician explained the technical aspects, prepared and advanced the Mentimeter slides, and safeguarded against facilitator biases.

While we did not set up a formal framework to collect participants’ opinions and feedback, we naturally observed and received many reactions as part of the feedback loop between facilitators and students. We summarised the most important observations.

### Data analysis

Group and individual thresholds from aNGTs were calculated directly by dividing the weight given to false positives by the total weight. CIs at the group level were calculated empirically based on the individual thresholds under the normality assumption, given that no systematic deviations from this assumption were observed.

Group and individual thresholds from the vignettes were calculated by performing a logistic regression of the decision to take action (yes=1, no=0) as a response against the probability of disease associated with the vignette as the sole predictor. The threshold was the probability of disease for which the probability of taking action was equal to 50%, equivalent to a log-odds of 0. CIs were calculated using the bootstrap method. The probability of RR-TB (in patients with confirmed TB) for the vignettes was estimated through expert consensus. Four TB experts studied each vignette, independently provided a probability of RR-TB and then discussed the results, reaching a consensus for each vignette. The probability of COVID-19 was estimated based on a simplified version of an unpublished early outbreak dataset.

All data analyses were done in R V.4.3.0. Bootstrapping was done using the boot package.^27^

### Patient and public involvement

Due to the retrospective nature of the study and its purpose as a pilot, no explicit patient and public involvement took place at this stage. As they are relevant stakeholders, patient and public involvement should be included in follow-up research on evaluating the feasibility and acceptability of the method.

## Results

### Participants

All participants were postgraduate students with experience in the healthcare sector. Due to the anonymity of the data collection, we do not have the demographic information of the participants. In table 1, the information for the students who enrolled in the course is summarised instead. On average, 90% of these students participated in the aNGT session. The median age was 35 years and all students were between 27 years and 50 years old. We strive for a gender balance in our classes to the extent possible. The majority of students work and live in Africa. Most students have an MD or MBBS and about half followed additional postgraduate education.

**Table 1:** Demographics of postgraduate students eligible to join an aNGT session

	RR-TB	Second-line HIV	COVID-19	Total
Enrolled in class	62	28	16	106
Participated (n (%))	55 (89)	25 (89)	15 (94)	95 (90)
Age (median (range))	35 (27–50)	36 (27–45)	37 (28–45)	35 (27–50)
Gender (n (%))				
Female	30 (48)	11 (39)	5 (31)	46 (43)
Male	32 (52)	17 (61)	11 (69)	60 (57)
Continent (n (%))				
Europe	10 (16)	2 (7)	2 (13)	14 (13)
Africa	33 (53)	22 (79)	13 (81)	68 (64)
Asia	17 (27)	3 (11)	1 (6)	21 (20)
Americas	2 (3)	1 (4)	0 (0)	3 (3)
Degree (n (%))				
MD/MBBS	60 (97)	22 (79)	15 (94)	97 (92)
Continued education*	35 (56)	13 (46)	3 (19)	51 (48)

*Any postgraduate degree other than MD/MBBS, for example, MPH, MSc, specialisation, PhD.

aNGT, adapted nominal group technique; RR-TB, rifampicin-resistant tuberculosis.

### Rifampicin-resistant tuberculosis

The main results from the RR-TB sessions are summarised in table 2. Using aNGT, the estimated thresholds at which one would start RR-TB treatment were similar across the different groups, at approximately 50%. This means that, on average, our panels would treat for RR-TB if the perceived probability of RR-TB is higher than 50%. However, each group had substantial individual variation, as illustrated by the relatively wide CIs. When the weighing step was repeated, the threshold did change, although interestingly, after the repeated weighing, it ended up more in line with thresholds from other sessions (of note, the thresholds of the other sessions were not known at the time that the weighing step was repeated in online session 1 was done).

**Table 2:** Action thresholds to give RR-TB treatment rather than RS-TB treatment, with 95% CIs, estimated with aNGT and clinical vignettes

Session	Participants*	#FP harms	#FN harms	aNGT threshold	Vignettes threshold
F2F 1	19	9	5	50.9% (43.4%–58.5%)	35.9% (29.0%–42.8%)
F2F 2	19	5	3	50.1% (43.2%–57.0%)	Not done
Online 1†	8	7	4	60.7% (57.2%–64.3%) 51.8% (45.6%–57.9%)	36.1% (29.6%–42.6%)
Online 2†	9	7	4	53.1% (44.3%–61.8%)	

*The number of participants refers to the number that was present in the aNGT sessions and provided weights.

†Weighing was planned to be repeated in both online sessions, but not done in online session two due to time constraints. The first percentage of online 1 is the result after the first weighing, the second shows the result after the weighing step was repeated.

aNGT, adapted nominal group technique; F2F, face to face; FN, false negative; FP, false positive; RR-TB, rifampicin-resistant tuberculosis; RS-TB, rifampicin-susceptible TB.

The estimated thresholds through the use of clinical vignettes, were found to be 36% and were very similar between both groups. This is lower than the thresholds estimated with aNGT. Therefore, when confronted with patient profiles, less proof of RR-TB is needed to start RR-TB treatment than when all harms are considered and discussed with peers.

### Second-line HIV treatment

The main results from the HIV sessions are summarised in table 3. In both sessions, the thresholds are below 50%, with participants giving more weight to wrongly not switching compared with wrongly switching. While in the online session, the difference is quite small with a threshold between 40% and 50%, in the F2F setting the harm of wrongly not switching is considered to be approximately three times higher (75.7% vs 24.3%) leading to a lower threshold to switch. Repeating the weighing step did not substantially change the estimated threshold.

**Table 3:** Action thresholds to switch to second-line treatment for people living with HIV in a resource-constrained setting

Session	Participants	#FP harms	#FN harms	aNGT threshold
F2F	12	4	7	24.3% (20.1%–28.6%)
Online*	13	7	6	45.6% (41.6%–49.6%) 41.5% (38.8%–44.1%)

The weighing step was repeated in the online session.

*The first percentage of online is the result after the first weighing, the second shows the result after the weighing step was repeated.

aNGT, adapted nominal group technique; FN, false negative; FP, false positive.

### COVID-19 isolation

The main results from the COVID-19 session are summarised in table 4. The threshold estimated with aNGT is again considerably higher than the one estimated through clinical vignettes. On average less proof is needed to isolate a hypothetical person than when the associated harms of such decision are discussed and weighed against each other. Similar to the HIV example, both thresholds are below 50%. The harm of wrongfully not isolating (false negative) is considered higher than the harm of wrongfully isolating (false positive). This is despite the participants identifying more distinct harms associated with wrongfully isolating (5 vs 3), which got a lower weight than the fewer but more heavily weighted harms associated with wrongly not isolating.

**Table 4:** Action thresholds to isolate people who may be infected with SARS-CoV-2 very early in the pandemic in a low-resource setting

Session	Participants	#FP harms	#FN harms	aNGT threshold	Vignettes threshold
F2F	15	5	3	43.3% (36.9%–49.8%)	24.3% (18.6%–30.0%)

aNGT, adapted nominal group technique; F2F, face to face; FN, false negative; FP, false positive.

### Facilitator observations

Reactions were majority enthusiastic, with participants finding the mix of voting and group discussion to work towards a consensus, while discussing and learning about the important harms, typically a very positive and enlightening experience.

When confronted with the average weights, shown in Mentimeter, and the associated threshold, participants usually found the resulting weights sensible with comments typically focused on rather small differences in opinion for a single harm. Participants always agreed when asked if they'd accept the consensus threshold for programmatic implementation if it would have been a real expert meeting, even when their individual thresholds showed considerable variation.

## Discussion

We developed and piloted a new method to estimate action thresholds in medicine. Unlike any existing method, our method takes into account all harms, including those that cannot easily be quantified. Additionally, it allows the relevant stakeholders to debate and work towards a consensus.

Due to the absence of a gold standard, it is impossible to verify if the thresholds are accurate. Nevertheless, results are precise enough to be applied in clinical practice and confidence widths similar to those obtained from clinical vignettes.

For RR-TB, a case study found that the threshold in Mozambique was possibly close to, but lower than 47%, but with large uncertainty.^21^ This is close to the thresholds that we found with either method. For the HIV example, while the results are quite different in the two sessions, both are in line with the expert consensus reached in a relevant setting.^19^ For COVID-19, it should be noted that either of the estimated thresholds would have been insufficiently low to slow down an exponential increase in cases.^24 25 28^ However, given that the example posited an early outbreak scenario in a low-resource setting when the infectiousness of SARS-CoV-2 was not yet well known, this threshold seems reasonable. Moreover, in hindsight, COVID-19 had considerably less impact on the healthcare system in sub-Saharan Africa^29 30^ while the economic burden of lockdowns was and would have been substantial.^31^

However, due to the experimental nature of the study, there are many limitations, and this pilot can only be interpreted as a proof of concept of the method.

First and foremost, the method should now be tested on professionals and stakeholders in a variety of settings where the action threshold is unknown and consensus guidelines need to be developed. While our participants were generally motivated and took the exercise seriously, some limitations were observed. The majority of participants were medical doctors with research and/or clinical experience, although rarely in managerial roles. With the exception of the COVID-19 example, none of them was a (former) patient, making the panels not sufficiently representative. The estimated thresholds should therefore be handled with caution.

For all examples, despite being specialists in their own right, there was a wide variation in relevant knowledge among the participants, ranging from a basic professional understanding of the topic to multiple years of experience in the field dealing with patients. As a learning objective, it was beneficial that knowledge could be exchanged through the group discussion, but for the sake of this pilot, it cannot be ignored that some answers may have been biased. This was more obvious in the clinical vignettes where for a few participants, there was no obvious relation between the probability of disease and the desire to take action. Additionally, agreement with the final action threshold could be due to desirability bias, although facilitators did create an environment in which critical remarks were welcomed. Creating such an environment in a professional setting may be harder, especially if there is a power imbalance, a history of one-sided or individual decision-making, or insurmountable differences in opinion between the programme manager and clinicians.^17 32 33^

One could argue whether a single true threshold exists, even when the setting is specified. Prescriptive methods, such as aNGT, weigh the harms against each other and as such define an ideal theoretical threshold based on the von Neumann-Morgenstern utility theorem. However, such thresholds may overlook or inaccurately weigh the different harms or utilities involved. While in theory, all harms are quantifiable, computing the true utility or harm of some is likely an intractable problem (eg, stigma, psychosocial consequences, future compliance, …). Hence, intuitive estimation of their relative weight compared with other harms may be the only feasible solution in practice. If experts and stakeholders would reach a consensus on these weights, we argue that we should accept their experience in the field given that they are the ones who take responsibility for these decisions on a daily basis and have the most complete view of the harm induced by wrong decisions, even if some harm may be perceived and thus weighed higher, or lower by them than a theoretical model would point it out to be.

Nevertheless, humans may be prone to a lot of cognitive biases. Therefore, for aNGT to work well, it is critical that all harms are included and properly discussed, and that everyone is sufficiently critical of other people’s biases, and their own. One recurring example is the power of the story of a patient. Some harms, such as stigma and income loss, can be illustrated by participants recalling compelling examples of real patients. This can potentially result in higher weights, while other harms such as transmission may be undervalued due to their theoretical nature, especially in decision-making for a single patient. Conversely, in the HIV example, it could be seen that considering fewer harms for a false positive impacted the threshold estimate. It can be hypothesised that if a facilitator brought up additional harms, participants would have given them some weight, thus increasing the threshold. Additionally, the facilitator can influence results through anchoring and adjustment bias, for example, through unequal time allocation to the different types of harm or pursuing opinions of only those who gave a specific (eg, low) rating or weight to a harm.

Additionally, it is of utmost importance to remember that the total harm is the product of the severity and the probability of that harm occurring. We noticed in the discussion several instances of the base rate fallacy and zero-risk bias, where participants, for example, strongly argued about the importance to avoid death even when the actual probability of death occurring when the wrong action is taken would be relatively small compared with the other harms. On the other hand, transmission was sometimes argued to be unavoidable, even when taking action would drastically reduce transmission. When only the severity of the harm is considered, and not its probability, results will be biased.

Descriptive methods such as the vignettes may not provide an estimate of the ideal threshold but instead match actual thresholds resulting from guidelines or expert opinions that are already in place. In addition to directly inheriting all biases from a potentially imperfect decision-making process that led to the guidelines, social desirability bias may play a role when people answer these. Since thresholds from guidelines can be directly calculated when the diagnostic accuracy of any symptom or test is known, such a method is only useful if participants are told explicitly to answer what they would like to do if there were no guidelines in place.

It was somewhat surprising to us that the thresholds estimated with the vignettes were systematically lower than those estimated with aNGT. One possible explanation is the adage ‘first do no harm’ may have a larger impact during group discussion, resulting in a higher weight for false positives. It is unclear if this is also a true difference in the actual threshold, that is, whether clinicians are less likely to take action in case of doubt when they have the opportunity to confer with colleagues.

## Conclusion

We developed a new and promising method for estimating action thresholds in medicine. However, further research is needed to study the value of the method in clinical decision-making and its ability to generate acceptable thresholds that relevant stakeholders can agree on.

## Data Availability

Data are available on reasonable request. All outcome data are available on reasonable request. Please contact the corresponding author and/or itmresearchdataaccess@itg.be.
