# Supplementary material for: Piloting a new method to estimate action thresholds in medicine through intuitive weighing
Source: BMJ Evid Based Med. 2023 Aug 30;28(6):392–8. doi: 10.1136/bmjebm-2023-112350 (PMC10715543; doi:10.1136/bmjebm-2023-112350)
Supplement: Supplementary data [file bmjebm-2023-112350supp001.pdf]

Piloting a new method to estimate action thresholds in medicine through intuitive weighing – supplementary file

Bart K.M. Jacobs[1*], Alfred Kipyegon Keter[1,2,3], Aquiles Rodrigo Henriquez-Trujillo[4], Paco Trinchan[5], Madeleine L. de Rooij[1], Tom Decroo[1], Lutgarde Lynen[1]

1. Institute of Tropical Medicine, Antwerp, Belgium
2. Ghent University, Ghent, Belgium
3. Human Science Research Council, Pietermaritzburg, South Africa
4. Universidad de Las Américas, Quito, Ecuador
5. Medical Officer. Bulawayo City Council, Bulawayo, Zimbabwe

Full example of an aNGT – RR-TB treatment (F2F 1)

Step 0 – introduction

The participants are first introduced to the topic of clinical decision-making and the relevant problem for which an action threshold is needed. This can be done both through an example (would you treat this case for which the diagnosis is unclear?) and by explaining the concept of action thresholds.

In our example, we combined a PowerPoint presentation, group discussion, an example of a real case and a short exercise to introduce these concepts to the postgraduate students. When time is limited, this can be limited to a short presentation.

Step 1 – generating and summarising harm statements

Once all participants understand the concept, the method itself (aNGT) and the platform used to collect the data (in our case Mentimeter™) are introduced. Participants are shown the first slide, asked to go to menti.com and instructed on how to provide their answers. Screenshots of what is shown publicly and what participants see on their device are shown in Figure S1.

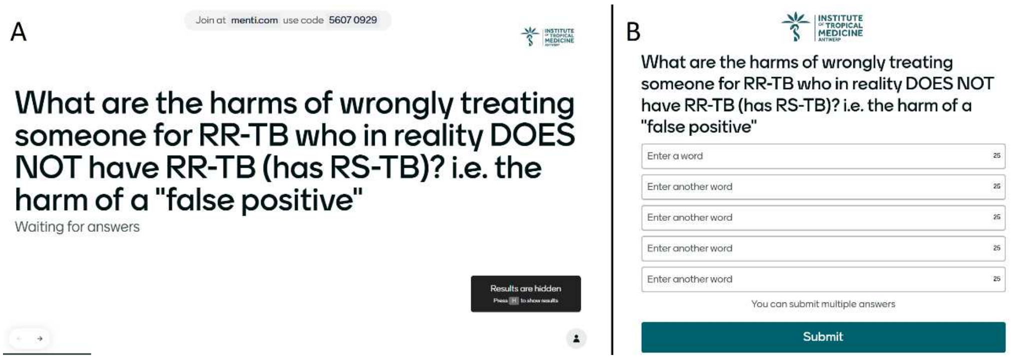

Figure S1 On the left (panel A) is what is shown publicly and, on the right, (panel B) is what is seen on the participants' screens during the first step of elicitation of the harms of the false positive clinical decisions for the RR-TB example.

Afterwards, the results are shown to the participants using a word cloud, as shown in Figure S2. Under the guidance of the facilitator, the harms are then summarised in mutually exclusive agreed-upon terms that are clear for all participants. E.g. For the false positives, both "adverse events" and "toxicity" refer to the same underlying harm, which is the side-effects of the more toxic second-line

regimen compared to the first-line regimen. In this session, participants decided to describe this as "toxicity and its consequences", shown as the first harm in Figure S3.

The same is then done for the harms of false negative clinical decisions, for which the resulting word cloud is also shown in Figure S2.

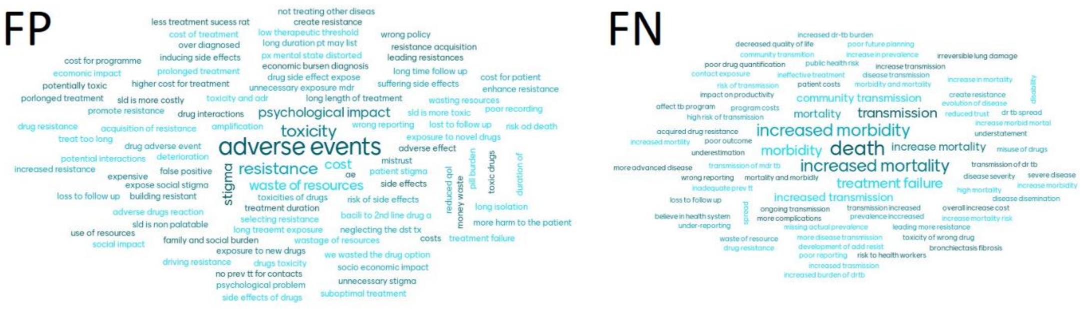

Figure S2 Word cloud of the different harms reported by the participants, for the false positive (FP) (left panel) and for the false negative (FN) (right panel) clinical decisions

Step 2 – Rating and discussing harm statements

The summarised statements are then rated anonymously using the same Mentimeter™ presentation as in the first step. We used a modified Likert scale for this purpose, although in essence any scale can be used with two main features: (1) the result can be shown as a distribution of how important participants find it to avoid the harm when taking their treatment decision and (2) it challenges participants to think about the relative importance of the harms without the answer given to one harm necessarily directly impacting the answer given for other harms. In the example shown here, we used a six-point Likert scale ranging from 0 to 5, with possible interpretation

- 0 – not a factor that needs to be considered
- 1 – a factor that should be considered but not given much weight in the treatment decision
- 2 – a reasonably important factor that may influence the treatment decision
- 3 – an important factor that will typically influence the treatment decision
- 4 – a very important factor with a major impact on the treatment decision
- 5 – a most important factor which will be a driving force in the treatment decision

The summarised harms and answers are shown in Figure S3. We alternated between false positives and false negatives to reduce any impact of the order. Instead, we focused on the distribution of the given scores and elicited discussion based on these.[1] In practice, the harms were considered one by one and participants were asked if they wanted to expound on why certain scores (i.e. rather high, rather low, in the middle, …) were given. It was allowed but never requested to disclose one's own rating. Consequently, participants regularly used language such as: "I rated this statement rather high because (…)". Arguments were typically a mix of published scientific facts and general knowledge (e.g. mortality rates), explanations on the impact in the setting of the participant (e.g. impact of personal cost on families and communities) and anecdotal evidence (e.g. examples of past patients who suffered greatly from a harm). Naturally, statements with more variation in the ratings evoked more discussion. Within the available time, facilitators tried to allow everyone to express their thoughts while also working towards a consensus-minded understanding of all participants. A

[1] Mentimeter™ also provides an average rating for each statement based on the corresponding numeric input. Since it is not good practice to calculate and interpret an average on a Likert scale, we choose to ignore this number.

consensus did not need to be a middle ground in this case. Sometimes, after discussion, people agreed that the harm was closer to the higher or lower end of the votes because arguments brought to the table by one or more participants were very convincing.

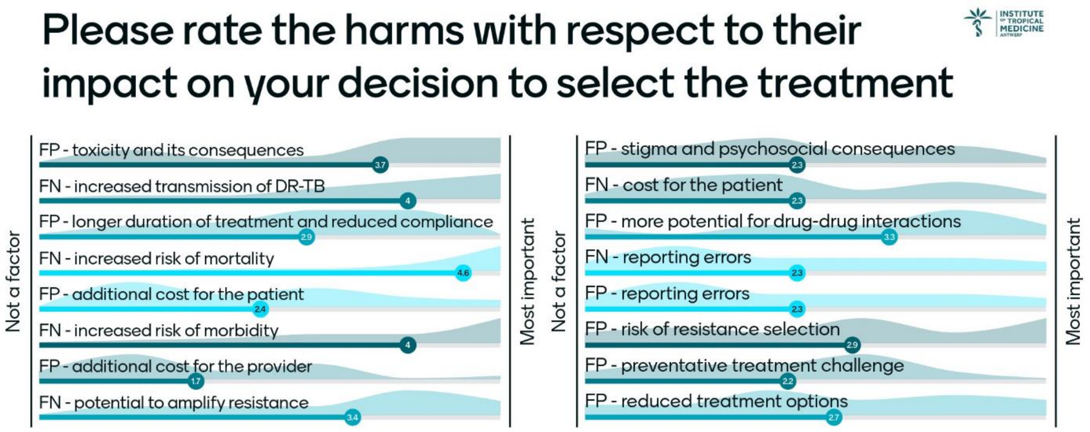

Figure S3 The resulting ratings as they are shown in Mentimeter™ (2 separate slides merged)

To check if there is more consensus after the discussion, the rating step can be repeated (without additional discussion), but this was not done in this specific example due to time constraints.

In this specific example, we see that participants identified considerably more distinct harms for false positives (10) versus harms for false negatives (6), but rated the false negative harms typically higher (although no formal tests for difference in rating were done). Increased risk of mortality, the increased risk of morbidity and the transmission of RR-TB were the highest rated harms overall, while toxicity was the highest rated harm among the false positive harms.

For several harms, including cost and stigma, the rating is not for the actual cost and stigma, but for the net difference between this harm for RR-TB treatment versus RS-TB treatment.

Usually, all harms rated and discussed are then included in the weighing step, unless it becomes clear that harms are in reality not taken into account in treatment decisions. This was the case here for reporting errors which were subsequently excluded from the weighing step.[2]

Step 3 – Weighing harm statements and estimating the action thresholds

In the last step, the participants are asked to weigh the statements against each other by distributing 100 points, corresponding to 100 percentage points. This is the hardest part for most participants, leading us to gradually increase the number of instructions that we give. In the most recent aNGTs, an additional slide (shown in Figure S4) was explicitly shown, while in earlier aNGTs similar instructions were given, yet only verbally.

[2] The group discussion revealed that a large majority of participants felt that the harms of reporting errors cancel each other out. Additionally and most critically, none of the participants truly took these harms into account in actual treatment decisions or believed that they should be taken into account in future treatment decisions. Indeed, the discussion shifted towards the importance of quality control and better reporting guidelines (at which point the facilitators had to move things along for the sake of time management), which goes beyond the clinical decision making process. This implies that the actual weights assigned to reporting errors is indeed 0 and they can be removed. Careful consideration that the true weight is indeed 0 (or negligibly close to 0) is always required before removing a harm from the weighing step.

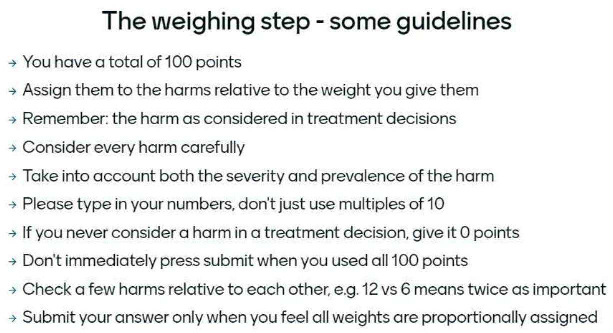

Figure S4 Instructions given to participants when distributing the weights in more recent aNGTs

After all participants had submitted their weights, the final results were displayed publicly using the slide included here as Figure S5. This shows the average of the weights given by all participants, rounded to the nearest integer and sorted from highest to lowest. Due to the rounding, it is possible for the displayed weights to fail to add up to exactly 100%, but in the underlying raw data, they do. Note that the lengths of the bars in Figure S5 are more exact and not rounded, with for example different lengths of bars for the harms that have an equal rounded weight of 4%. At this stage, repeating the weighing is possible as well, but that was neither planned, nor done during this session.

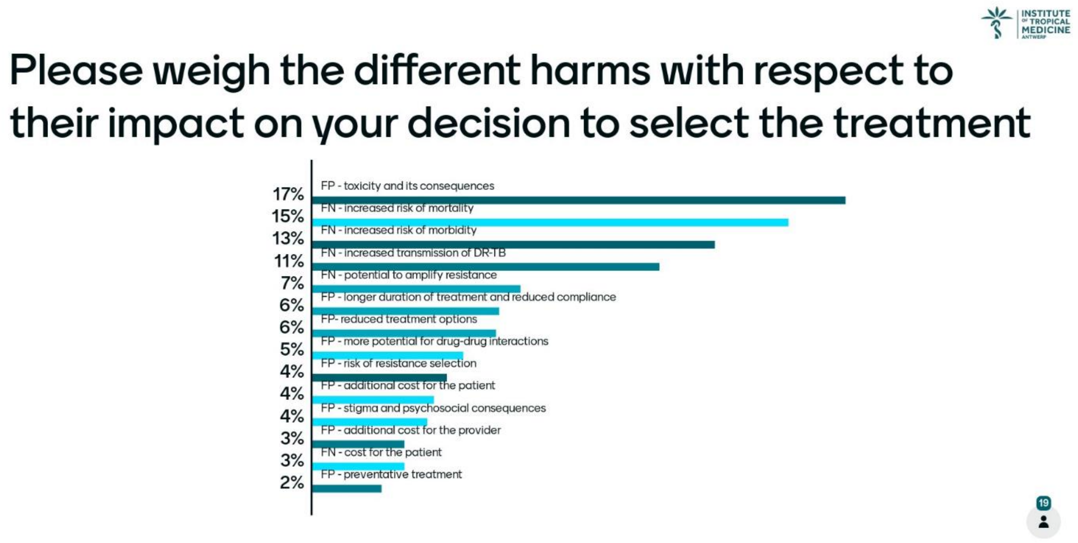

Figure S5 Average weights of the harms given by the participant

As this was in the first place also a teaching tool, we typically gave the participating postgraduate students some time to digest the results, discuss whether they agreed with them and then asked if they could derive the action threshold from them.

The harm of toxicity ended up being given the most weight on average, with the three highest rated harms from the previous step closely behind. In line with the rating step, the false negative harms were given higher weights despite being fewer in number. In this specific case, the percentages do sum up to 100% exactly, with the weights for the false positive harms summing up to 51% and the false negatives to 49%. For the results presented in the main document, we used the raw data of all

the participants and obtained an estimate of 50.9%. The variation around each average and the confidence interval around the estimated action threshold can also be obtained using the raw data.

Multiple harms that are either hard to, or cannot be quantified theoretically are given substantial weight. This includes increased transmission, the potential to amplify resistance, reduced compliance and reduced future treatment options. At best, any formal EUT estimate for these harms would be very imprecise and depend on unverifiable modeling assumptions.

Step 4 – conclusion

The aNGT ends here, with the estimated threshold being announced to all participants. In our sessions, we added additional discussions on the interpretation and potential implementation of this threshold in practice, and in sessions where students provided answers to the clinical vignettes (like the one in this example), results were compared to the estimates obtained from the clinical vignettes before closing the session.
